# Detecting early gastrointestinal polyps in histology and endoscopy images using deep learning

**DOI:** 10.3389/frai.2025.1571075

**Published:** 2025-07-08

**Authors:** Intissar Dhrari Hajsalem, Yassine Ben Ayed

**Affiliations:** ^1^National Engineering School of Sfax (ENIS), University of Sfax, Sfax, Tunisia; ^2^Multimedia, Information Systems and Advanced Computing Laboratory (MIRACL), University of Sfax, Sfax, Tunisia

**Keywords:** deep learning, gastrointestinal cancer, ROI detection, feature extraction, detection polyps

## Abstract

**Introduction:**

The GastroIntestinal Cancer (GIC) is one of the most common tumors in terms of deaths and diseases. Artificial Intelligence (AI) domains such as Deep Learning (DL) have the potential to greatly improve the early identification of disease. Nevertheless, a lot of current technologies are still insufficient to detect tumors, which is why we created an approach using advanced method to identify polyps.

**Methods:**

Our three-stage deep learning-based method requires constructing an Encoder-Decoder Network (EDN) to determine the Region of Interest (ROI) in preprocessing, feature selection with pretrained models such as VGG16, VGG19, ResNet50 and InceptionV3, and Support Vector Machine (SVM) classifier to separate affected individuals from normal ones during the classification stage. Five datasets, such as CRC-VAL-HE-7K, CRC-VAL-HE-100K, Kvasir_v2, a dataset from Beijing Cancer Hospital, and a weakly labeled dataset, containing histology and endoscopic images, were utilized to train and evaluate our method.

**Results:**

The outcomes showed the effectiveness of our approach, with these pretrained models obtaining the best efficiency for recognizing gastrointestinal polyps. ResNet50 attained the maximum accuracy on datasets 1, 2, and 4, with performances of 97.01%, 96.49%, and 98.90%, respectively. Also, VGG16 and VGG19 performed 96.64% and 98.75% accuracy on datasets 3 and 5, respectively. However, InceptionV3 scored slightly less well than the other model.

**Discussion:**

The advanced method produced promising results for the early detection of gastrointestinal cancer in multiple datasets.

## 1 Introduction

The prevalence of gastrointestinal diseases has increased each year, especially gastrointestinal cancer, which is one of the most prevalent illnesses that impact a large number of people every day. Many studies in the healthcare field have discovered that consuming too much meat, not enough fruits and vegetables, eating unhealthy meals, and consuming alcohol and tobacco are a number of the causes to this cancer. Gastrointestinal cancer is a very dangerous that affected the gastrointestinal system form esophagus into the anus, and is common throughout the world. According to scientific statistics, there are 4.1 million new incidents of GIC and almost three million individuals die from this disease (Kuntz et al., 2021). In this context, there are many groups that can be classified into this category of cancer, which include colorectal, esophageal, gastric, pancreatic, and liver cancers. First, colorectal cancer, which places third worldwide in terms of new cases and is the second most prevalent cause death (Azar et al., 2023). Next, gastric cancer (Loddo et al., 2024) sits fourth internationally in terms of mortality and fifth in terms of new infections. In addition, esophageal cancer stands seventh in terms of incidence and sixth in terms of mortality in the world (Lin et al., 2024). Additionally, an important proportion of the global population is affected by pancreatic and liver cancers.

Furthermore, the use of Computer-Aided Diagnostic (CAD) systems is necessary in correcting a variety of problems associated with manual detection, such as consuming time and many errors. CAD systems are useful in the early detection of gastrointestinal cancer and recognizing polyps.

Deep Learning is an advanced artificial intelligence method that promises improved diagnosis accuracy by detecting gastrointestinal polyps using a variety of techniques and lower the death rate linked with these diseases. Also, it is an excellent method compared to hand-crafted techniques.

Moreover, DL, which uses several layers of a neural network, aims to extract and select significant features. Convolutional Neural Networks (CNNs) are highly specific for segmenting and rapidly identifying important features in histological, endocscopy, and Magnetic Resonance Imaging (MRI) images. Their effectiveness in this field is demonstrated by their ability to identify gastrointestinal cancer.

In this research, we propose a CAD system that uses five distinct datasets to identify gastrointestinal polyps. Three steps compose our system: preprocessing the data, extracting features from the polyps, and a supervised model, namely support vector machine, is used in the classification step, after selecting the most important and relevant features. Our contributions in this research are outlined by the following:

- Propose a method to detect the region of interest in cases of gastrointestinal cancer applying an encoder-decoder network.

- Evaluating deep pre-trained models' output to extract pertinent and significant features and select the ideal model for each dataset.

- Develop a classification method to improve our approach to identifying and recognizing gastrointestinal cancer.

This paper presents a comprehensive analysis of the most commonly used segmentation and detection techniques, offering a detailed investigation into early gastrointestinal cancer diagnosis. In Section 2, we discuss the state of the art, which defines methods for polyp segmentation such as encoder-decoder and transfer learning for feature selection. Furthermore, in section 3 we describe our proposed approach for identifying gastrointestinal polyps. Thereafter, in section 4, we discuss our experimental research, in which we conducted various tests to evaluate our approach with the different datasets using evaluation metrics. The section 5, titled “discussion,” provides a summary of our efforts. Finally, Section 6 reviews our work and our proposed approach to identifying gastrointestinal tumors to conclusion.

## 2 Related works

This section presents several investigations that use the encoder-decoder network for polyp segmentation, pretrained models for feature extraction and classification. First, we report a studies obtained to extract and identify the region of interest of polyps. A technique to detect Lymph Node (LN) regions using a U-shaped Neural Network (U-Net) was presented by Wang et al. (2021b). Next, in order to detect polyps, TS and Jagadale (2023) compared U-Net and DeepLab segmentation methods. In comparison to DeepLab, which obtained an Intersection over Union (IoU) of 0.9676, they reported a UNet IoU of 0.9897, showing a consistent and accurate polyp segmentation, applying the CVC-ClinicDB dataset. In the same context, Dumitru et al. (2023) introduced a novel approach called “DUCK-Net: Deep Understanding Convolutional Kernel” based on the architecture of UNet with the aim to identify tumors, utilizing Kvasir-SEG, CVC-ClinicDB, CVC-ColonDB, and ETIS-Larib PolypHD datasets. Subsequently, utilizing the CVC-EndoSceneStill and Kvasir-SEG datasets, Sánchez-Peralta et al. (2020) applied U-Net and data augmentation to recognize polyps. In addition, Azar et al. (2023) employed a data augmentation technique. Lastly, Lin et al. (2024) proposed a novel approach termed no-new-Net (nnU-Net) for the segmentation of esophageal cancer.

The related works on feature extraction and classification for gastrointestinal cancer are presented in the second part of this section. In comparison with the handcrafted technique, the approaches for Deep Learning highlighted the important attribute that ensures a good performance for segmenting and detecting pertinent information. According to Kuntz et al. (2021), a convolutional neural network was applied for the early detection of gastrointestinal cancers. In this context, the research by Azar et al. (2023) introduced a CNN-based technique for colon cancer detection using variation optimizers: Adam, Nadam, SGD, Adadelta, RMSprop, and Adamax. They used the CRC-VAL-HE-7K and Warwick-QU datasets of histological images, to achieve this goal. They reported that CNN with Adam optimizer obtained a greater accuracy of 90.00% for the CRC-VAL-HE-7K dataset, while the accuracy for the Warwick-QU dataset was 76.00%. The authors in Wang et al. (2021b) compared models to select the most appropriate one for extracting important features, including VGG19, AlexNet, ResNet-18, ResNet-34, ResNet-50, ResNet-101, Inception V3, Inception V4, and MobileNet V2 and for classifcation they used a neural conditional random forest.

Next, Uddin et al. (2024) developed an approach that uses several models, such as DenseNet201 and ResNet50, for feature extraction and fully-connected layers for classification. These researchers used the NCT-CRC-HE-100K and CRC-VAL-HE-7K datasets, which contain histological images to classify colon and colorectal cancer. They also applied various scenarios, and the best accuracy value was 96.26%. In addition, Sun et al. (2023) proposed a new system that attempts to detect three distinct forms of cancers, including colorectal cancer, They employed fully-connected layers to categorize benign and malignant categories. After using the pre-trained model ResNet50 to identify a significant characteristic. To accomplish this objective, the authors utilized NCT-CRC-HE-100K for training and CRC-VAL-HE-7K for testing and they achieved an accuracy of 94.80%. Then, the purpose of the study (Nogueira-Rodríguez et al., 2021) was to examine the most pertinent tasks in the context of deep learning models, such as ResNet-50, VGG16, VGG19, AlexNet, GoogLeNet, Fast CNN, InceptionV3, Inception-ResNet-v2, and ResNet-101. Thereafter, Lee et al. (2024) created a technique to detect a Lymphovascular Invasion (LI) in gastric cancer implementing variours of models, ResNet50, EfficientNetB3, ConViT, YOLOv3, and YOLOX. The highest accuracy 93.53%, was achieved by YOLOX.

Furthermore, Su et al. (2022) developed a new system capable to identify the tumor and Microsatellite Instability (MSI) in gastric cancer. It consists of two crucial phases: the diagnosis of tumors and the detection of microsatellite instability. For this objective, they introduced ResNet-18 for extraction feature and fully-connected layers for classification. The dataset used is based on data from the Peking Cancer Hospital, obtained from 2015 to 2020. In identifying MSI, they reported an accuracy of 86.36%. In the same context, Park et al. (2023) implemented a number of models, including InceptionNet-V3, EfficientNet-B7, VGG-16, ResNet-50, DenseNet-121 and ViT to diagnose digestive diseases, considering the Kvasir_v2 dataset. With an accuracy of 94.90%, InceptionNet-V3 provided the best results. The work by Chughtai et al. (2024) developed a novel method named DeepCon, based on pre-trained models Xception and InceptionV3 to collect features and fully-connected layers to classify colorectal cancer.The CRC-VAL-HE-7K dataset, which has 5,000 photos split into 8 classes with 625 images for each class, is used for this proposed approach. The model Xception outperformed the others, obtaining an accuracy and an F-measure of 98.20%.

Also, Loddo et al. (2024) presented a method to classify histological images of stomach cancer into groups of normal and malignant, using GasHisSDB dataset. During the “feature extraction” step, there are two distinct categories of features: handcrafted features, which are comprised of color features, texture features like Local Binary Patterns (LBP), and invariant moments. The second group's deep feature is composed of ResNet (types 18, 50, 101), XceptionNet, VGG19, DenseNet-201, ResNet-v2, Inception-v3, Inception-EfficientNetB0, DarkNet, and AlexNet (types 19, 53), They employed Decision Trees (DT), Random Forests (RF), support vector machines, and k-Nearest Neighbors (kNN) in the classification phase. They also documented that LBP performed better than other methods with all four classifiers (SVM, kNN, DT, and RF) for handcrafted feature extraction, whereas DenseNet-201 yielded the best results for deep feature extraction. The best outcome was obtained by combining the SVM classifier with LBP, DenseNet-201, and EfficientNetB0, yielding an accuracy rate of 95.03% and an F1 score of 95.90%.

In addition, the study (Lin et al., 2024) was published to differentiate cancer patients from non-cancer cases using a decision tree. This method demonstrated a high level of diagnostic efficacy in the detection of esophageal cancer, with a precision of 88.20% and an F-score of 89.10%. Using the NCT-CRC-HE-100k dataset (80% for training, 20% for validation), the CRC-VAL-HE-7K dataset (for testing), the TCGA-CRC and SYSU-165 datasets, Qi et al. (2021) utilized VGG19 to capture features from histopathological images of colorectal cancer. They showed that this model generated a 95.00% accuracy rate. Furthermore, Shen et al. (2023) created a CNN-based model to identify characteristics, and they classified colorectal polyps using EfficientNet-b0 applying endoscopic images from the Kvasir dataset. For polyp detection, they observed a specificity of 97.09% and a sensitivity of 97.01%, and for polyp classification with a sensitivity of 97.78% and a specificity of 98.89%. Moreover, Wang et al. (2021a) utilized an InceptionV3 model with fully connected layers to detect colorectal cancer. For this work, they employed a number of datasets: CRC-VAL-HE-7K, NCT-CRC-HE-100k, TCGA-Frozen, TCGA-FFPE, and SYSU-CGH. They reported accuracy was >90.00%.

For the diagnosis of colorectal cancer using histological images, the CNN model HCCANet was proposed by Zhou et al. (2022). It was based on a CAD system incorporated with an attention mechanism called MCCBAM (Multi-Channel and Channel-Spatial Block Attention Module). Data augmentation techniques were applied in the preprocessing phase. The experiment findings showed that HCCANet surpassed a number of advanced models, such as ResNet50, MobileNetV2, Xception, and DenseNet121, and also traditional models like K-nearest neighbors, naive bayes, random forest, and support vector machines. The accuracy of MCCBAM was 87.30%, which was the greatest when compared to other popular attention mechanisms which comprises SAM, SENet, SKNet, Non_Local, CBAM, and BAM. The pretrained model HCCANet performs exceptionally well in this situation, mostly because of its attention mechanism, which improves VGG16's potential to extract and identify essential features.

Finally, Khan et al. (2024) created a deep learning model based on histopathological images from the LC25000 dataset for colon cancer to categorize benign and malignant instances. First, in the preprocessing step, they applied a segmentation method to detect ROI and data augmentation methods like flipping and rotation to create more images. In the second step, they compared various models, notably ResNet-101, Swin Transformer, Swin Transformer with a Modified Last Layer, and Vision Transformer (ViT), to find pertinent information in colon polyps. After feature extraction, a fully connected layer was used in the third step to distinguish the cases as malignant or benign. Additionally, they showed that the various models obtained the following precision: 98.27% with ResNet-101, 99.36% with Vision Transformer (ViT), 99.64% with Swin Transformer (Modified Last Layer), and 99.80% with Swin Transformer. They reached the conclusion that the model with the best performance is Swin Transformer.

In conclusion, we observed that a variety of datasets containing histology, endoscopy, and MRI images are included in the field of computer vision for gastrointestinal cancer detection. These investigations employed several deep learning and machine learning models, showing promising outcomes in segmentation, classification, and recognition of different types of this cancer.

## 3 The proposed method

**Figure 1:**
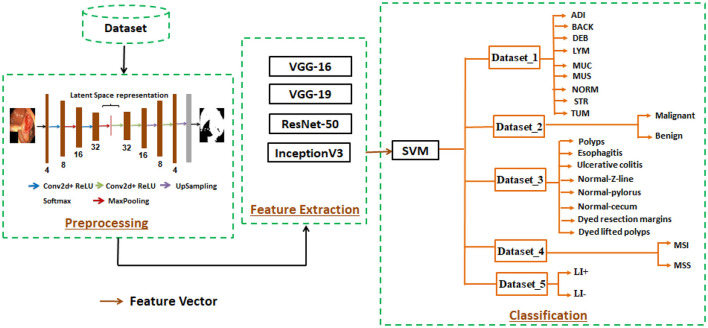
Descriptive representation of the proposed approach.

Our proposed method for detecting gastrointestinal polyps is based on deep learning techniques. It consists of three main phases: preprocessing, feature extraction, and classification. The first step, is to detect ROI using an encoder-decoder network, which selects relevant and significant information. By removing irrelevant information and efficiently identifying pertinent features, this method saved time and produced encouraging results for our proposed system in diagnosing gastrointestinal cancer. The second phase, is to find descriptive features by training pre-trained models like VGG16, VGG19, ResNet50, and InceptionV3. These models all have different layers and unique architectures. In the third phase, an SVM classifier is used to identify affected persons from normal persons, which is configured with an ideal combination of the cost parameter C, gamma parameter, and alternative kernels. We examined five different datasets (dataset 1, dataset 2, dataset 3, dataset 4, and dataset 5) in our efforts for evaluating our proposed approach. The three steps of our suggested approach are illustrated in Figure 1.

### 3.1 Preprocessing

In our research, we applied preprocessing, which is a crucial stage aimed at identifying and detecting the region of interest from the original image using advanced methods. Moreover, the identification of the ROI helps in detecting significant features, reducing processing time, and eliminating unnecessary information. This advanced technique has demonstrated beneficial results in polyp segmentation.

For this purpose, we propose a novel encoder-decoder network, since it can accurately segment and find ROI in medical images. When compared to traditional methods, it also exhibits greater effectiveness in segmentation tasks. Furthermore, it performs better at identifying quickly growing gastrointestinal polyps than other deep learning architectures that depend on a little simpler encoder-decoder structures and their objective to detect important information in polyps.

This developed approach consists of three phases. The first phase is the encoder, which isolates the latent representation of the input (original) image using a sequence of four 2D convolution layers with ReLU activation and two 2D MaxPooling layers. The second phase is the latent space, which comprises essential and pertinent data. The architecture of the encoder produces this representation of the latent space. Lastly, the decoder uses the latent space representation to produce a version that is exactly the same as the original image (input). The architecture of the decoder consists of a series of two UpSampling layers, five 2D convolution layers, and ReLU activation layers. Within this “encoder-decoder network,” several layers contribute to achieving effectiveness, described as follows :

- Convolutional layer : enables feature selection via filters.
- MaxPooling layer : after convolutional layer, we used MaxPooling layer, which aims to decrease the input image's size while highlighting the most significant characteristics.
- ReLU activation function : this function preserves positive values constant while converting negative values to zero.
- UpSampling layer : after the convolutional layer, we also use the UpSampling layer in the decoder architecture to optimize the representation's spatial dimensions.
- Softmax activation function : the decoder architecture is completed by the Softmax, which acts as the output layer.

An EDN configuration is defined and described in Table 1.

**Table 1:** The architecture parameters for ROI detection using EDN.

**Layer**	**Type**	**Number of filters**	**Filter size**	**Stride**	**Activation**
**L1**	Conv	4	3 × 3	1 × 1	ReLU
**L2**	Conv	8	3 × 3	1 × 1	ReLU
**L3**	MP	-	2 × 2	1 × 1	ReLU
**L4**	Conv	16	3 × 3	1 × 1	ReLU
**L5**	Conv	32	3 × 3	1 × 1	ReLU
**L6**	MP	-	2 × 2	1 × 1	ReLU
**L7**	Conv	32	3 × 3	1 × 1	ReLU
**L8**	Conv	16	3 × 3	1 × 1	ReLU
**L9**	US	-	2 × 2	1 × 1	ReLU
**L10**	Conv	8	3 × 3	1 × 1	ReLU
**L11**	Conv	4	3 × 3	1 × 1	ReLU
**L12**	US	-	2 × 2	1 × 1	ReLU
**L13**	Conv	3	3 × 3	1 × 1	Softmax

Conv, Convolutional; MP, MaxPooling; US, UpSampling.

### 3.2 Feature extraction

In this stage, we used a comparative analysis to identify the best model and the one that could extract descriptive data. For this work, a number of models were trained using the five datasets (Dataset 1, Dataset 2, Dataset 3, Dataset 4, and Dataset 5), including VGG16, VGG19, ResNet50, and InceptionV3.

We utilized the VGG16 model, proposed in 2014 by Simonyan and Zisserman (2014), which consists of 16 layers. This architecture includes 13 2D convolution layers with ReLU activation, five 2D MaxPooling layers, three fully connected layers, and one softmax layer. We also employed the VGG19 model, introduced by Simonyan and Zisserman (2014) from the VGG research group. Compared to VGG16, this model has 19 layers more blocks than VGG16. Three fully connected layers, five 2D MaxPooling layers, sixteen 2D convolution layers with ReLU activation, and one softmax layer are all part of this architecture. Similarly, the ResNet-50 model, created in 2015 by He et al. (2016), is characterized by the use of residual connections. This pretrained model's architecture contains 50 layers, comprising 48 2D convolution layers, one Max Pooling layer, and one Average Pooling layer. Additionally, we utilized InceptionV3, established by Szegedy et al. (2016), which is an extension of another model. Forty-eight layers with a variety of operations make up its architecture. Because of their excellent results in terms of identifying and detecting pertinent and crucial traits, we decided to work with these models The architecture of pre-trained models VGG16, VGG19, ResNet50, and InceptionV3 used these different layers convolutional, pooling (such as MaxPooling, Global Average Pooling), dropout, fully connected, and an output.

- Convolutional layer : this layer is the first in the architecture of all models and requires to extract significant characteristics using filters.
- Max pooling : this layer is utilized for these various pretrained models and chooses the highest value in each input region to perform successful feature extraction.
- Global average pooling : this layer calculates the average value of each feature map. It is applied in the architecture of ResNet50.
- Fully connected layers : after the “Flatten” layer, the fully connected layer analyzes the input (image) and groups it into different categories based on its characteristics.
- Dropout layer : in order to reduce overfitting, neural networks employ the dropout layer as a method of regularization before the fully connected layers.
- Output layer : the output layer is the final layer of a pre-trained model that generates outputs based on the collected features.

**Figure 2:**
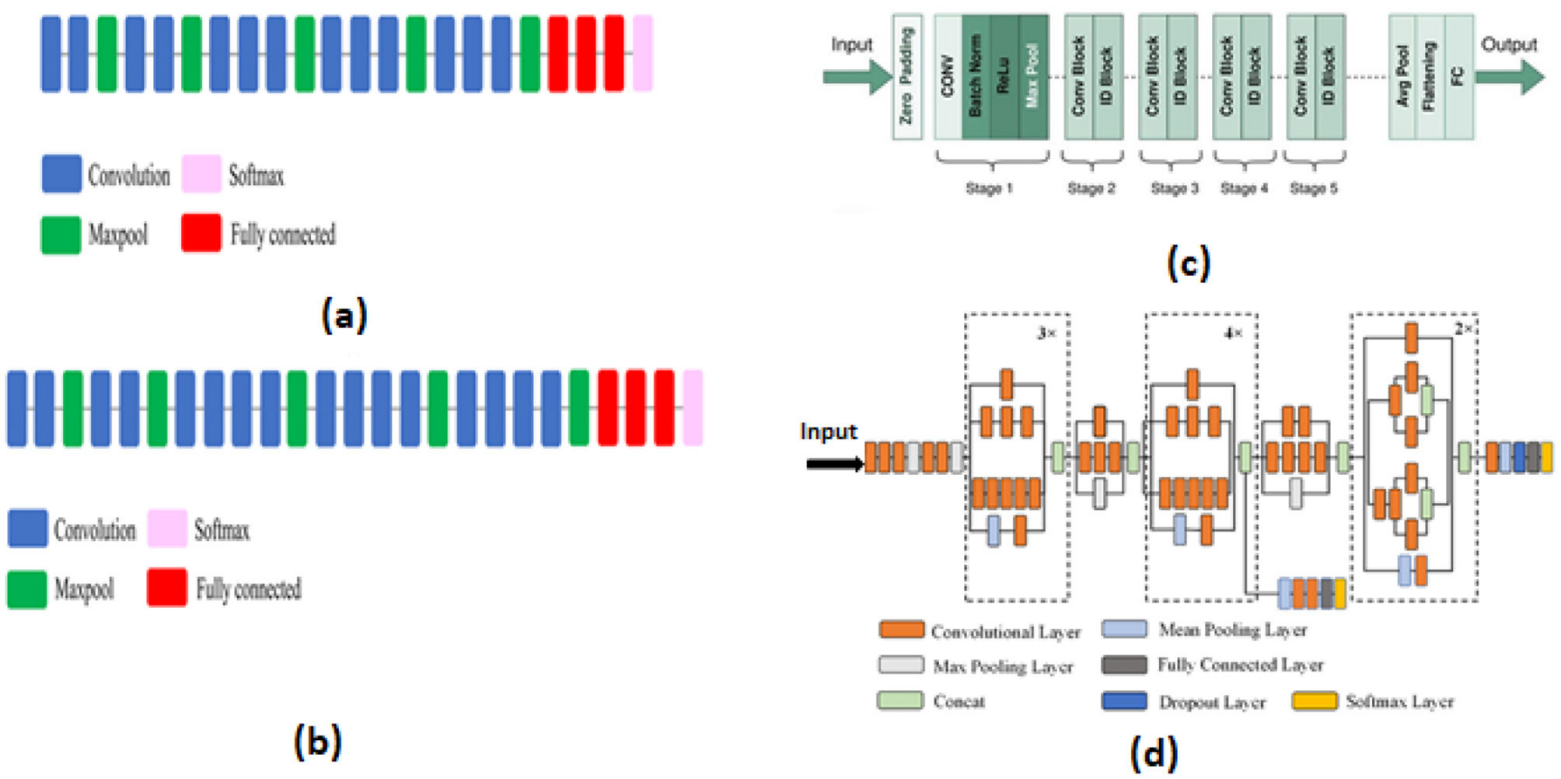
Structures of various models.

In this context, the evaluation of performance for these pre-trained models shows that ResNet50 achieves the highest accuracy on dataset 1, dataset 2, and dataset 4, with performances of 97.01%, 96,49%, 98.90% respectively. In the same context, VGG16 performs the best in dataset 3, achieving an accuracy of 96.64%. Lastly, VGG19 achieves an accuracy rate of 98.75% in dataset 5, demonstrating remarkable efficiency. We observed that the pre-trained model InceptionV3 is less effective compared to the other models, but ResNet50 demonstrates excellent performance in detecting pertinent information. The various model structures are shown in Figure 2. This diagram indicates that (a) corresponds to VGG16, (b) to VGG19, (c) to ResNet50, and (d) to InceptionV3, and each model has a distinct architecture.

### 3.3 Classification

In our proposed system, classification is the last stage. This stage uses the feature vector, which has significant and descriptive characteristics, a resulting from the pre-trained models, to differentiate between people who are healthy and those who have gastrointestinal cancer. In this context, we compared the two classifiers support vector machines and MLP “MultiLayer Perceptron.” The results showed that the SVM performed better at 98.00% accuracy, whereas the MLP did not surpass 75.00% accuracy.

Consequently, we decided to use SVM for our system. After validating our choice, we defined the SVM classifier, which is an automated technique that works to identify the perfect hyperplane. To separate data into their appropriate groups, this classifier uses three distinct kernels: a linear kernel, a polynomial kernel, and a radial basis function (RBF) kernel. These kernels produce good performance in a variety of methods.

## 4 Experimental results

### 4.1 Dataset

In our study, we utilized five different datasets (Dataset_1, Dataset_2, Dataset_3, Dataset_4, and Dataset_5) which contained histology and endoscopic images with 224 x 224 pixel, with 80% of the data for training and 20% for testing.

- **Dataset_1 :** This dataset, called “The Colorectal Cancer Validation Histology 7K” (CRC-VAL-HE-7K) (Azar et al., 2023), has 7,180 colorectal cancer histology pictures. These pictures, which come in nine categories (ADI, BACK, DEB, LYM, MUC, MUS, NORM, STR, and TUM), were taken from 50 patients.
- **Dataset_2 :** This dataset named CRC-VAL-HE-100k (Sun et al., 2023), which is similar to CRC-VAL-HE-7K, contains 100,000 images classified into nine classes (ADI, BACK, DEB, LYM, MUC, MUS, NORM, STR, TUM) from 86 individuals. These images are used to diagnose colorectal cancer.
- **Dataset_3 :** Kvasir_v2 (Park et al., 2023) is the name of this dataset, which contains a total of 8,000 images grouped into eight classes, with each class comprising 1,000 JPEG-encoded images. The classes are colored-lifted polyps, colored-resection margins, polyps, ulcerative colitis, normal-cecum, normal-pylorus, and normal-Z-line.
- **Dataset_4 :** The Beijing Cancer Hospital created this data between 2015 and 2020 (Su et al., 2022), which is used to diagnose microsatellite instability and tumors of gastric cancer. This dataset contains whole-slide images stained with hematoxylin and eosin (H&E). There are 348 patients allocated for training and 88 individuals designated for testing.
- **Dataset_5 :** In gastric cancer, this dataset named “weakly labeled dataset” (Lee et al., 2024) is utilized to identify lymphovascular invasion. It contains 2,471 images annotated as LI+ (lymphatic invasion present) and LI- (lymphatic invasion absent).

### 4.2 Evaluation metrics

We studied the data and found promising outcomes for our strategy, focusing on the following evaluation parameters : Accuracy (1), Precision (2), Recall (3), and F1 Score (4).


(1) $$\begin{array}{l} \text{Accuracy}=\frac{TP+TN}{TP+TN+FP+FN} \end{array}$$

(2) $$\begin{array}{l} \text{Precision}=\frac{TP}{TP+FP} \end{array}$$

(3) $$\begin{array}{l} \text{Recall}=\frac{TP}{TP+FN} \end{array}$$

(4) $$\begin{array}{l} \text{F1 score}=\frac{2\cdot \text{Precision}\cdot \text{Recall}}{\text{Precision}+\text{Recall}} \end{array}$$

The confusion matrix, which shows the results of predictions in an ordered matrix format, is composed of four variables : False Positive (FP), False Negative (FN), True Positive (TP), and True Negative (TN).

### 4.3 Results analysis

In the context of our research on gastrointestinal cancer detection, we analyzed the outcomes of several experiments using five different datasets (Dataset_1, Dataset_2, Dataset_3, Dataset_4, and Dataset_5). We studied the effectiveness of pretrained models like VGG19, VGG16, ResNet50, and InceptionV3 for every dataset. To find the ideal configuration of the SVM, we selected the gamma parameter at 0.01 and performed a variety of values for the cost parameter, including C = 10, C = 100, C = 1,000 and C = 10,000, with the objective of identifying the optimal value of C, which was C = 10,000. SVM comprised linear, polynomial, and RBF kernels were employed in the same context to assess the performance of this system.

In addition, we used this arrangement during the training process. We experimented with different combinations for the batch size and the total number of epochs. The ideal parameters were 300 for the batch size and 500 for the number of epochs. As a loss function, we use the Mean Squared Error (MSE) 5.


(5) $$\begin{array}{l} \text{MSE}=\frac{1}{n}\overset{n}{\underset{i=1}{\sum }}{\left(y_{i}-{ŷ}_{i}\right)}^{2} \end{array}$$

**Figure 3:**
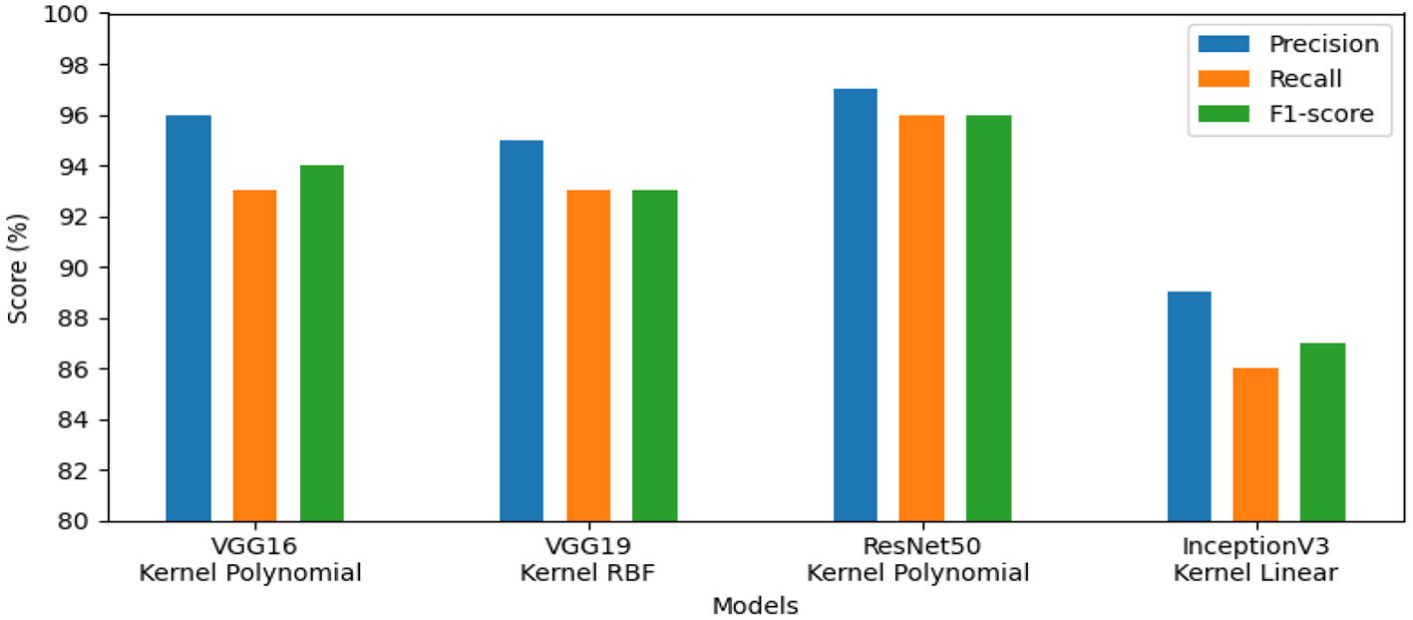
Results of the most favorable measures for dataset_1.

**Figure 4:**
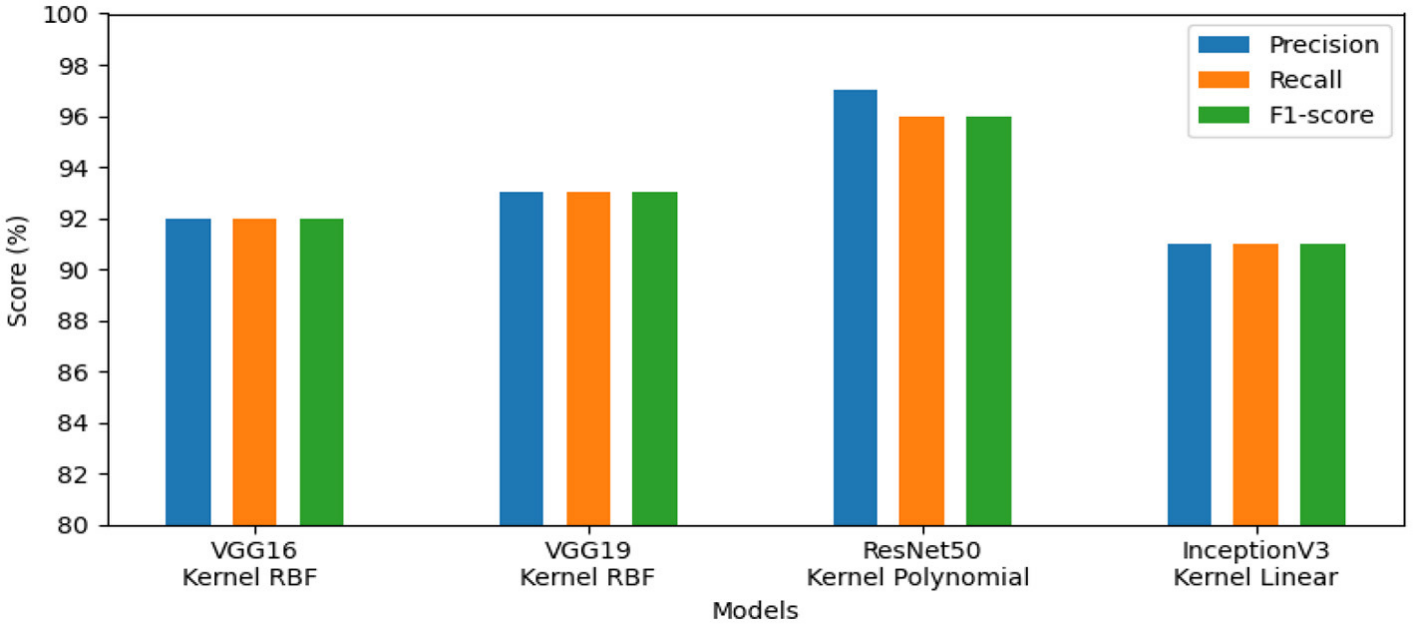
Results of the most favorable measures for dataset_2.

**Figure 5:**
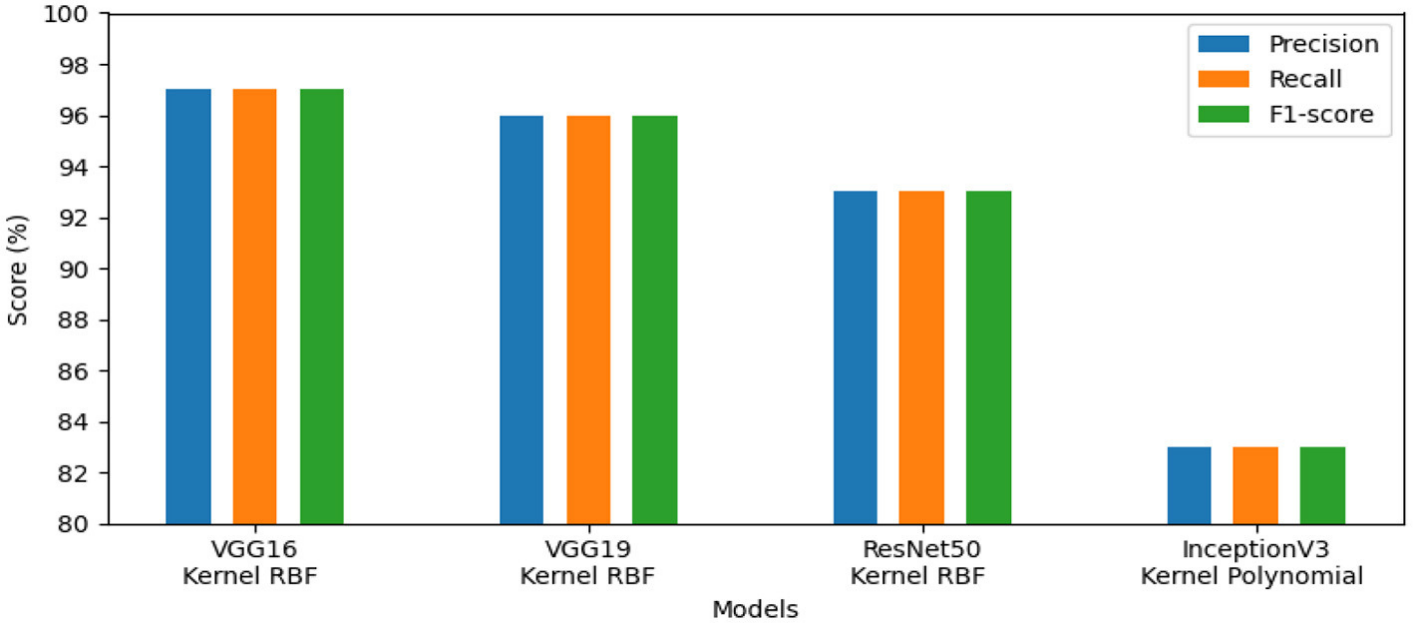
Results of the most favorable measures for dataset_3.

**Figure 6:**
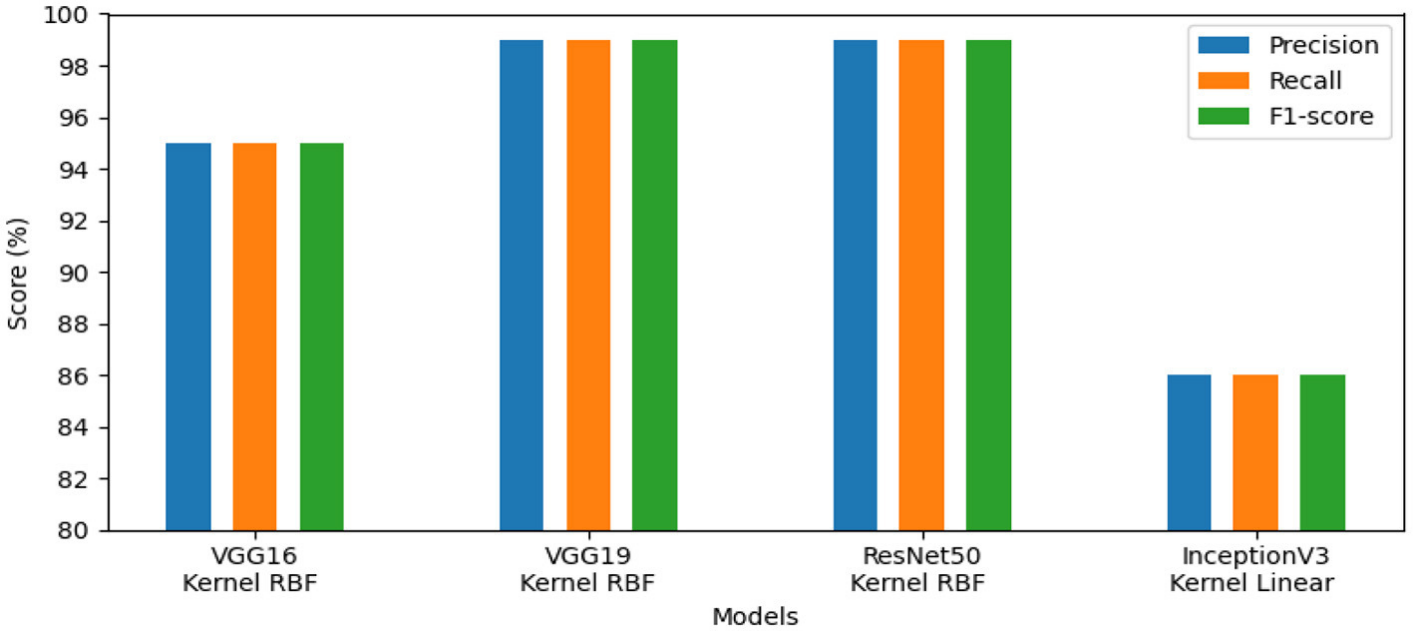
Results of the most favorable measures for dataset_4.

- The first set of experiments aims to assess and validate our method's for detection colorectal cancer performance using dataset_1 (CRC-VAL-HE-7K). We chose to take into account the nine classes that were represented in this analysis: ADI, BACK, DEB, LYM, MUC, MUS, NORM, STR, and TUM. As can be observed from the results in Table 2, the following represents the optimal accuracy for each of the pre-trained models VGG16 offers 94.79%, VGG19 has 94.37%, InceptionV3 has 87.50%, and ResNet50 provides the best accuracy with this dataset at 97.01% using the polynomial kernel. In the same context, Figure 3 illustrates the performance of the models using various metrics for Dataset_1. For the evaluation metric precision, VGG16 obtained 96.00%, VGG19 achieved 95.00%, ResNet50 reached 97.00%, and InceptionV3 scored 89.00%. In terms of recall, VGG16, VGG19, ResNet50, and InceptionV3 achieved 93.00%, 93.00%, 96.00%, and 86.00%, respectively. Lastly, VGG16, VGG19, ResNet50, and InceptionV3 received the corresponding scores of 94.00%, 93.00%, 96.00%, and 87.00% for the F1-score. In conclusion, ResNet50 produces the most effective results for feature selection on Dataset_1 in our system.
- To determine the effectiveness of our approach, we compare the performance of VGG19, VGG16, ResNet50, and InceptionV3 using two classes, malignant and benign. For training, we used the dataset_2 (CRC-VAL-HE-100K), and for testing, we employed the dataset_1 (CRC-VAL-HE-7K). Table 3 presents the outcomes of the various models. ResNet50 outperformed the other models, utilizing the polynomial kernel to obtain an accuracy of 96.49%. VGG19 achieved 93.20%, VGG16 obtained 92.49%, and InceptionV3 had 90.68%.
- The efficiency analysis demonstrated that the VGG16, VGG19, ResNet50, and InceptionV3 models achieved excellently. VGG16 scored 92.00%, VGG19 obtained 93.00%, InceptionV3 produced a 91.00% result on various evaluation metrics, However, ResNet50 achieved a precision of 97.00% and a recall and F1-score of 96.00%. For this dataset_2, ResNet50 is the best pre-trained model for feature extraction in our system. Figure 4 presents the performance models on various metric. In summary, ResNet50 outperforms the other models in terms of results for the two datasets.
- This experiment aims to detect polyps in the digestive system using 8 classes from the dataset_3 named kvasir-v2, which consists of endoscopic images. Table 4 provides the findings of our suggested approach, which show that VGG19 achieved 96.64% accuracy, VGG16 reached 95.96%, ResNet50 achieved 93.09%, and InceptionV3 obtained 83.45%. We concluded that VGG19 is the most effective model for feature extraction on this dataset after assessing each model's performance.
  The results of the four pre-trained models across various metrics are shown in Figure 5. VGG19 achieved 96.00%, whereas VGG16 reached 97.00%. Moreover, ResNet50 obtained 93.00%, and InceptionV3 achieved 83.00%. Our proposed method offers promising results in evaluation metrics such as precision, recall, and F1-score when using VGG16 for dataset_3.
- The objective of this experiment is to demonstrate the performance of our system in detecting microsatellite instability in gastric cancer using dataset_4 which contains two classes: MSI and Microsatellite Stability (MSS). The performance of pre-trained models for MSI recognition is shown in Table 5. In comparison, VGG19 achieved excellent accuracy of 98.86% with the RBF kernel. However, ResNet50 also preformed effectively achieving 98.90% with the kernel RBF, VGG16 came in second with 94.63%; and InceptionV3 came the last with 85.57%. Figure 6 displays the outcomes of the four pretrained models over a range of measures. VGG19 obtained 99.00%, while VGG16 obtained 95.00%. Additionally, InceptionV3 and ResNet50 both achieved 86.00% and 99.00%. Using ResNet50 for dataset_4, our suggested approach yields encouraging results in assessment measures like precision, recall, and F1-score.
- The goal of this present study is to recognize lymphovascular invasion in stomach cancer using dataset_5 which contains two classes: LI(+) (presence of lymphovascular invasion) and LI(-) (absence of lymphovascular invasion). We used data augmentation to correct the imbalance present in dataset _5. This method creates new photos by applying different modifications (rotation, scaling, flipping, and cropping) to the original images with the aim of increasing the dataset. Consequently, 4,000 images with these various arrangements were generated from 2,471 images.

**Table 2:** Performance of VGG19, VGG16, ResNet50, and InceptionV3 with dataset_1.

**Model**	**Accuracy (%)**		
	**Kernel linear**	**Kernel polynomial**	**Kernel RBF**
VGG16	94.37	**94.79**	93.54
VGG19	93.61	93.47	**94.37**
ResNet50	96.80	**97,01**	96.80
InceptionV3	**87.50**	86.25	84.16

**Table 3:** Performance of VGG19, VGG16, ResNet50, and InceptionV3 with dataset_2.

**Model**	**Accuracy (%)**		
	**Kernel linear**	**Kernel polynomial**	**Kernel RBF**
VGG16	89.00	83.00	**92.49**
VGG19	90.76	87.18	**93.20**
ResNet50	95.87	**96.49**	96.38
InceptionV3	**90.68**	89.90	85.43

**Table 4:** Performance of VGG19, VGG16, ResNet50, and InceptionV3 with dataset_3.

**Model**	**Accuracy (%)**		
	**Kernel linear**	**Kernel polynomial**	**Kernel RBF**
VGG16	95.02	95.40	**96.64**
VGG19	94.09	94.28	**95.96**
ResNet50	89.11	92.16	**93.09**
InceptionV3	80.52	**83.45**	83.39

**Table 5:** Performance of VGG19, VGG16, ResNet50, and InceptionV3 with dataset_4.

**Model**	**Accuracy (%)**		
	**Kernel linear**	**Kernel polynomial**	**Kernel RBF**
VGG16	93.15	92.70	**94.63**
VGG19	95.22	97.17	**98.86**
ResNet50	98.19	94.70	**98.90**
InceptionV3	**85.57**	82.83	85.33

The effectiveness of our method for detecting lymphovascular invasion is demonstrated in Table 6, where VGG19 outperforms all other models in our proposed approach, achieving an accuracy of 98.75%. This exceeds the accuracy of 98.62% for ResNet50, 98.38% for VGG16, and 98.00% for InceptionV3. Our proposed method confirms its effective performance in identifying gastric cancer with RBF kernel using this histology dataset.

**Table 6:** Performance of VGG19, VGG16, ResNet50, and InceptionV3 with dataset_5.

**Model**	**Accuracy (%)**		
	**Kernel linear**	**Kernel polynomial**	**Kernel RBF**
VGG16	97.50	98.00	**98.38**
VGG19	97.75	98.75	**98.75**
ResNet50	97.75	98.50	**98.62**
InceptionV3	96.38	97.88	**98.00**

**Figure 7:**
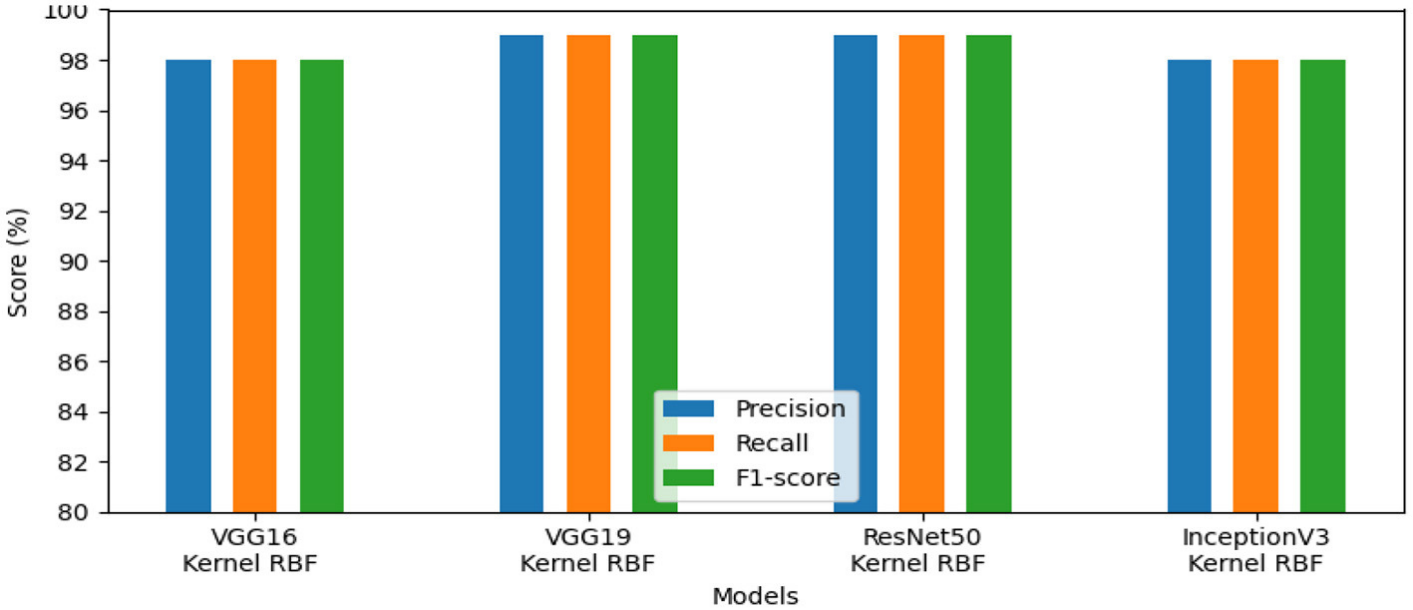
Results of the most favorable measures for dataset_5.

The models' performance assessed using a variety of criteria, comprising F1-score, precision, and recall and is illustrated by our system in Figure 7. InceptionV3 gained 98.00%, ResNet50 reached 99.00%, VGG19 attained 99.00%, and VGG16 achieved 98.00%. Our approach demonstrated exceptional performance for identifying lymphovascular invasion (LI) in dataset_5.

- To validate the quality of our proposed method in comparison to related research for the identification of gastrointestinal cancer. We present our findings in Table 7. We emphasize that dataset_1 is utilized in Azar et al. (2023), whereas dataset_2 is employed for training and dataset_1 for testing in Sun et al. (2023). Additionally, dataset_3 is used in Park et al. (2023). On the other hand, dataset_4 is used in Su et al. (2022), and dataset_5 comes from Lee et al. (2024). It is clear from a comparison of our performance results with the state-of-the-art methods that our proposed method produced the best outcomes. Specifically, using dataset_1 improved the accuracy by 7.01% compared to Azar et al. (2023). Using dataset_2 for training and dataset_1 for testing increased the accuracy by 1.69% as opposed to Sun et al. (2023). Additionally, in comparison with Park et al. (2023), utilizing dataset_3 improved accuracy by 1.74%. Moreover, our approach exhibited a significant accuracy improvement of 12.54% utilizing dataset_4, when compared to Su et al. (2022). Lastly, comparing dataset_5 results with those from Lee et al. (2024) optimized the accuracy by 5.22%.

**Table 7:** Comparative studies.

**Method**	**Type of dataset**	**Accuracy (%)**
Azar et al. (2023)	Dataset 1	90.00
**Proposed method**		**97.01**
Sun et al. (2023)	Dataset 2	94.80
**Proposed method**		**96.49**
Park et al. (2023)	Dataset 3	94.90
**Proposed method**		**96.64**
Su et al. (2022)	Dataset 4	86.36
**Proposed method**		**98.90**
Lee et al. (2024)	Dataset 5	93.53
**Proposed method**		**98.75**

Our approach confirms the success rate for the first ROI detection by employing an encoder-decoder network, which enhances the feature quality during the process of recognizing the pertinent and significant information. Additionally, selecting features with pretrained models such as VGG16, VGG19, ResNet50, and InceptionV3 and then classifying SVM to categorize groups from the dataset.

In summary, our CAD system is an excellent choice because it consistently produces the best results, without regard to changes in the dataset, the quantity of data, or the types of images.

## 5 Discussion

Our study has the potential to identify gastrointestinal cancer using different deep learning architectures. First, we employ deep learning method for polyp segmentation in endoscopic and histological images. Then, we used machine learning model for classification and pre-trained models for feature extraction.

The experimental analysis showed the effectiveness of our proposed approach for the early detection and diagnosis of gastrointestinal polyps from five distinct datasets dataset_1, dataset_2, dataset_3, dataset_ 4, and dataset_5.

Our automated system exhibited excellent accuracy for these datasets. The ResNet50 model produced good results on dataset_1, dataset_2, and dataset_4, attaining accuracy scores of 97.01%, 96.49%, and 98.90%, respectively. Using VGG16, dataset_3 obtained 96.64% accuracy, whereas dataset_5 used VGG19 to reach 98.75% accuracy. The performance of other metrics, such as precision, recall, and f1-score, also demonstrated encouraging results. For dataset_1, the evaluation metrics varied between 86.00% and 97.00%. In addition, the scored measures for dataset_2 comprised 91.00% to 97.00%. Dataset_3's assessment metrics ranged from 83.00% to 97.00%. Also, the evaluation measures for dataset_4 ranged from 86.00% to 99.00%. With final evaluation metrics for dataset_5 falling between 98.00% and 99.00%.

Moreover, the ROI detection, using an encoder-decoder network, confirmed the importance of optimizing data quality by selecting relevant information and reducing redundancy. After processing the data, we employed pre-trained models such as, VGG19, VGG16, Resnet50 and InceptionV3 for feature extraction, which each one of models offers a good results, and an SVM classifier was used to divide the dataset groups, demonstrating higher effectiveness compared to an MLP classifier.

In the final analysis, our method successfully detected gastrointestinal cancer, confirming the value of artificial intelligence in anomaly detection for medical imaging. Additionally, this work emphasizes the crucial role of deep learning and shows that the pre-trained models exhibit good results in improving the identification of gastrointestinal polyps. Then, advanced techniques, particularly CNNs, produce excellent results in computer vision.

**Figure 8:**
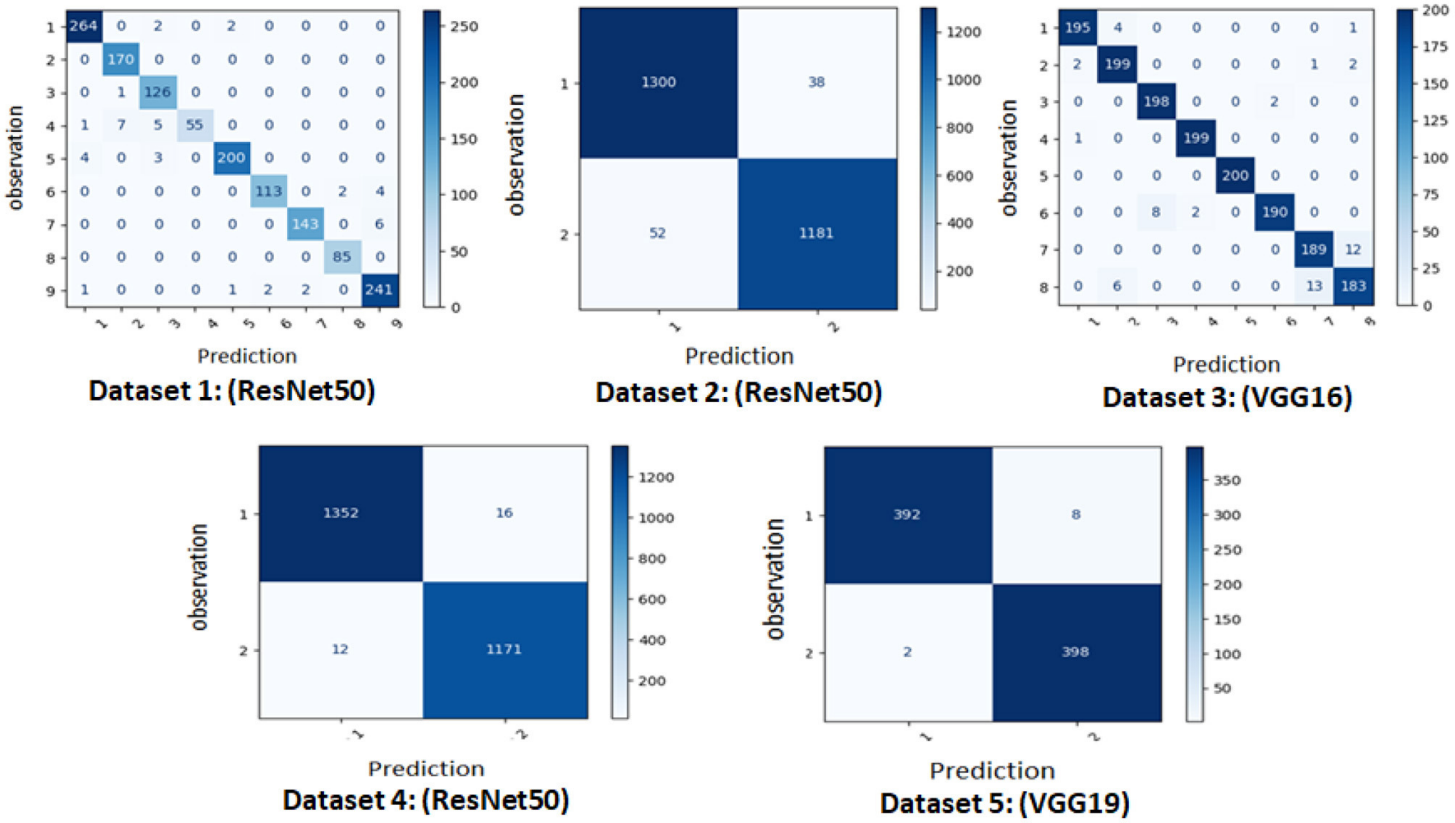
Confusion matrix of the best model for each dataset.

We show the top-performing model for each dataset in Figure 8. On datasets 1, 2, and 4, ResNet50 produced the best results. On dataset_3, VGG16 exhibited the best performance, while on dataset_5, VGG19 was the greatest model.

## 6 Conclusion

In this research, we developed a system for detecting gastrointestinal polyps based on deep learning. The region of interest is identified using an encoder-decoder network, a model designed for image segmentation that aims to detect significant regions within the image for preprocessing, pretrained CNN models are used for feature extraction as the suitable approaches for detecting and assessing polyps from data based on images, and finally, support vector machine is an automated classifier separates data into corresponding groups for classification. The discipline of computer vision encompasses the use of advanced methods for anomaly detection, and our proposed approach applies deep learning and transfer learning for recognizing this type of cancer.

Other deep learning architectures exist, therefore the suggested method is not the only strategy for identifying gastrointestinal cancer. Developing alternative architectures for preprocessing, feature extraction, and classification, and also validating our approach on additional diseases, are potential future projects.

## Data Availability

The original contributions presented in the study are included in the article/supplementary material, further inquiries can be directed to the corresponding authors.
